# Smartphone Apps for Cardiopulmonary Resuscitation Training and Real Incident Support: A Mixed-Methods Evaluation Study

**DOI:** 10.2196/jmir.2951

**Published:** 2014-03-19

**Authors:** Marco Kalz, Niklas Lenssen, Marc Felzen, Rolf Rossaint, Bernardo Tabuenca, Marcus Specht, Max Skorning

**Affiliations:** ^1^Welten Institute-Research Centre for Learning, Teaching and TechnologyOpen University of the NetherlandsHeerlenNetherlands; ^2^Department of AnesthesiologyUniversity Hospital AachenRWTH Aachen UniversityAachenGermany; ^3^Medical Advisory Service of Social Health InsuranceEssenGermany

**Keywords:** basic life support (BLS), cardiopulmonary resuscitation (CPR), external chest compression (ECC), smartphone apps, mobile phone, mobile health

## Abstract

**Background:**

No systematic evaluation of smartphone/mobile apps for resuscitation training and real incident support is available to date. To provide medical, usability, and additional quality criteria for the development of apps, we conducted a mixed-methods sequential evaluation combining the perspective of medical experts and end-users.

**Objective:**

The study aims to assess the quality of current mobile apps for cardiopulmonary resuscitation (CPR) training and real incident support from expert as well as end-user perspective.

**Methods:**

Two independent medical experts evaluated the medical content of CPR apps from the Google Play store and the Apple App store. The evaluation was based on pre-defined minimum medical content requirements according to current Basic Life Support (BLS) guidelines. In a second phase, non-medical end-users tested usability and appeal of the apps that had at least met the minimum requirements. Usability was assessed with the System Usability Scale (SUS); appeal was measured with the self-developed ReactionDeck toolkit.

**Results:**

Out of 61 apps, 46 were included in the experts’ evaluation. A consolidated list of 13 apps resulted for the following layperson evaluation. The interrater reliability was substantial (kappa=.61). Layperson end-users (n=14) had a high interrater reliability (intraclass correlation 1 [ICC1]=.83, *P*<.001, 95% CI 0.75-0.882 and ICC2=.79, *P*<.001, 95% CI 0.695-0.869). Their evaluation resulted in a list of 5 recommendable apps.

**Conclusions:**

Although several apps for resuscitation training and real incident support are available, very few are designed according to current BLS guidelines and offer an acceptable level of usability and hedonic quality for laypersons. The results of this study are intended to optimize the development of CPR mobile apps. The app ranking supports the informed selection of mobile apps for training situations and CPR campaigns as well as for real incident support.

##  Introduction

The pervasive use of mobile phones has motivated several initiatives to integrate them into the chain-of-survival for cardiac arrest [1]. While the phone has naturally been used to support bystanders remotely with dispatcher instructions, recently several initiatives have made use of the advanced capabilities of smartphones [2,3]. For a variety of reasons, the rate of bystander cardiopulmonary resuscitation (CPR) is low. In Germany, for example, the bystander CPR rate is approximately 20% [4]. Reasons often mentioned are a lack of knowledge and the fear to perform mouth-to-mouth ventilation [5,6]. This is one reason for the introduction of “compression-only CPR” in Basic Life Support (BLS) guidelines from the European Resuscitation Council (ERC)/American Heart Association (AHA). This easy-to-learn approach (“push hard and fast in the middle of the chest”) without ventilation is intended to alleviate fear and motivate more of the public to perform CPR. Smartphones have the great advantage of providing situational support and easily accessible information (ie, apps) and therefore have become more and more valuable for CPR training and real incident support.

A recent systematic review has shown that a variety of mobile health apps for medical professionals and patients is available [7]. Due to the high number of available apps, some authors even speak about a phenomenon of “app overload” [8]. Since there is no quality control on the content and usability of a mobile app, quality and conformity with guidelines cannot be guaranteed. The term “usability” has been defined as the ease of use and learnability of a human-made object, and usability design guidelines have been defined by the International Standardization Organization in the standard ISO/TR 16982:2002 [9]. Holzinger mentions five criteria—learnability, efficiency, memorability, low error rate, and satisfaction—as essential characteristics of usability [10].

How many and, in particular, which apps might be helpful in supporting cardiopulmonary resuscitation (CPR) training as well as in a real incident of cardiac arrest is unknown. A helpful app should include correct and current medical content and deliver this content with high usability. In this context, our study provides an overview of the quality of available mobile apps. We report results of a mixed-methods evaluation study of mobile training and real incident support apps for cardiopulmonary resuscitation. This study is part of the European project “EMuRgency - New approaches for resuscitation support and training” [11].

## Methods

### Design

**Figure 1:**
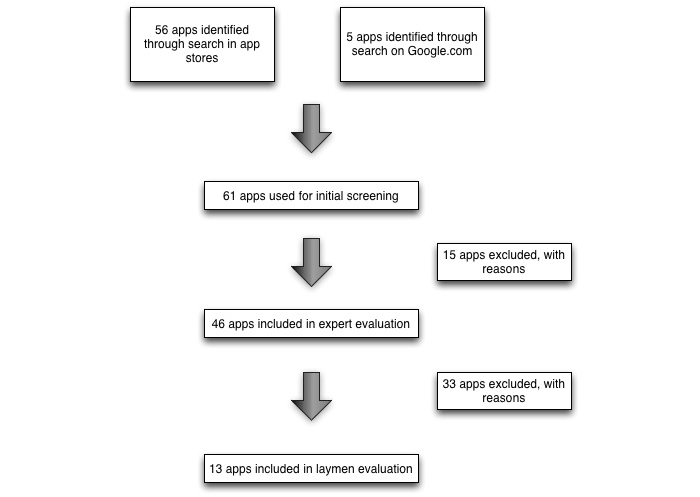
App selection procedure.

In this study, we applied a mixed-methods sequential design (Figure 1). Initially, an identification of apps and expert evaluation was conducted. As a second step, layperson users were involved to evaluate the usability and the appeal (“hedonic quality”) of the preselected apps. In the study, we followed guidelines for agreement studies [12].

### Participants

Six board-certified emergency physicians from Germany agreed on minimum medical content requirements for apps to be included in the evaluation phase. Two of them screened and evaluated the apps and 14 layperson volunteers were recruited from the staff of the Open University of the Netherlands for the second phase of the evaluation. The recruitment of the volunteers was organized via a news item on the intranet of the Open University.

### Materials

#### Overview

The material for the study consisted of mobile phones and mobile apps. Materials used in the expert evaluation were two smartphones (iPhone 4S & HTC Desire) equipped with the apps to be reviewed. In the second evaluation phase, three phones (iPhone 3S, iPhone 4S, and HTC Desire) were used in combination with an iPad and a computer to fill out the questionnaires. The identification and selection of mobile apps to be included in the study is reported based on PRISMA guidelines [13].

#### Identification of Apps

In May 2012, we conducted a search on the two largest online stores for mobile applications (Google Play Store and Apple App Store). Search terms were “CPR” and “resuscitation”. In addition, we conducted a Google search using search words “CPR apps” and “resuscitation apps”.

#### Screening

CPR apps containing Basic Life Support (BLS) and/or Advanced Life Support (ALS) material were considered eligible. At this stage, apps were screened and classified according to their different features, namely:

- type of content (video instructions, video chest compression simulation, animations, graphics, audio instructions, audio chest compression rhythm simulation, text instructions)
- aim of the app (training or real incident support)
- only CPR or several first aid features
- mobile sensors used in the app (GPS or accelerometer)
- underlying guidelines (most notably American Heart Association and/or European Resuscitation Council), date of guidelines (2010 or older)
- correct reproduction of guidelines’ recommendations
- targeted patient (adult, children, infant, animal)
- language (English, German, Spanish, or others)
- cost of the app
- mobile operating system (iOS or Android)
- company or provider of the app on the market

#### Eligibility/Inclusion

To our knowledge, no quality and/or content criteria for CPR apps have been defined to date. Due to broad approval within the resuscitation research group, the features shown in the first category in Table 1 were set as mandatory for inclusion in further evaluation steps with non-medical end-users. The feature in the second category was considered as important. The third category contains desirable special features. Hence, two board-certified emergency physicians screened the apps for requirements on these three quality levels.

**Table 1:** Requirements catalogue for mobile app screening by experts.

Mandatory features	Important feature	Special features
Training feature	Direct access to emergency call (112, 911, 999)	Focus on compression-only CPR
Conformity to ERC/AHA 2010 BLS Guidelines		Use of accelerometer
Emergency support for real incidents		

### Instruments

After identifying and screening the apps, experts’ opinions were sought in a first evaluation. Raters were prompted to take the following items into account:

- estimated benefit for users compared to conventional teaching material
- estimated usability (ease of use regarding the user interface and logic of handling the different parts of the app)
- feature quality (video/graphic/picture/animation/audio/text instructions, graphic/animation/audio/video beat rhythm)
- application possibilities (training, real scenario support, accelerometer, location GPS, direct access to emergency call number, includes compression only CPR)
- CPR focus
- consideration of current guidelines (American Heart Association, European Resuscitation Council)

Independently, two experts rated each app on an ordinal scale (0 to 10=unsatisfactory to perfect). Testing and rating time for each app was adapted to rater’s needs. In total, each expert completed eight test sessions. Each session lasted 2-4 hours. An interrater reliability analysis using Cohen’s kappa (weighted) was performed to determine consistency among both raters. The first phase of the evaluation focused on ensuring sufficient content quality, instructional value, conformity with current guidelines, and availability of a minimal set of features as listed above.

The second phase of the evaluation focused on usability and hedonic quality of the mobile apps.

For the usability evaluation, we used the System Usability Scale (SUS) [14]. This tool is a simple but reliable method to evaluate the usability of a diverse set of technologies [15,16]. The SUS scale consists of 10 questions with a 5-point Likert scale, where item directions are changed with each question. Results of the SUS questionnaire were recoded and normalized. A specific value of usability was calculated for each app. Based on current literature, a SUS score above 68 (SD 12.5) is rated as a usability value above average. To benchmark these results against other results, we followed recommendations by Sauro and converted raw SUS scores to percentile ranks [17]. This conversion maps the raw SUS results to results from 446 studies including over 5000 individual SUS responses. While a raw SUS score can theoretically be 100, the distribution of available SUS scores is negatively skewed and therefore the conversion in percentile ranks results in more meaningful results. A raw SUS score of 73 results in a percentile rank of 66.5%. This means that the object of evaluation can be considered more usable than 66.5% of all products evaluated with the SUS instrument.

We have calculated an intraclass correlation coefficient (ICC) for uneven and even questions (ICC1 and ICC2). The SUS scale has been used earlier to evaluate the usability of medical devices and mobile devices in a hospital context [18].

Hassenzahl has criticized that the current approaches to test usability have taken into account only the user’s recognition of design objectives represented by the ergonomic quality but not the subjective experience in terms of user satisfaction. To take the non-task-related quality dimensions like originality or innovativeness into account, he proposes the concept of “hedonic quality”, which represents the appeal of a user-interface [19]. Other authors have stressed the importance of appeal for the design of user interfaces and the potential negative consequences if technology is designed based only on a functional definition of usability [20].

**Figure 2:**
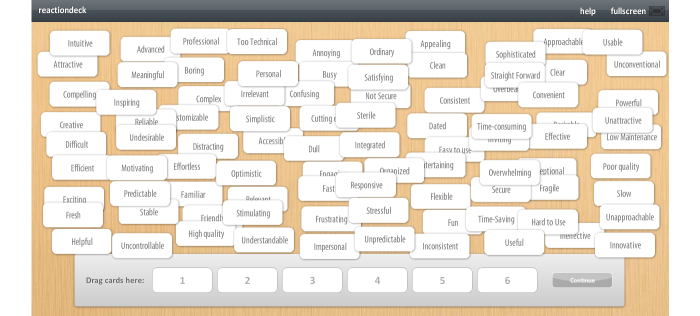
ReactionDeck toolkit.

To evaluate the hedonic quality, we employed the ReactionDeck toolkit (Figure 2). This toolkit is based on the desirability toolkit developed by Benedek and Miner at Microsoft Research to assess aspects like “desire” and “fun” of products [21]. These product reaction cards have been transferred to digital format by the Open University of the Netherlands and published as ReactionDeck toolkit [22]. Thus, participants were asked to select six product reaction cards that best describe the emotional appeal of the mobile applications.

Between December 2012 and February 2013, 14 evaluation sessions took place with volunteers. Each evaluation session lasted approximately 60 minutes and was conducted in a standardized evaluation laboratory setting.

Demographic details and experiences with resuscitation and mobile apps were collected with a pre-questionnaire. Apps were randomly assigned to participants. Participants were asked to use the respective mobile apps for learning basic cardiopulmonary resuscitation knowledge and skills and were thus assigned to them via one of the three study smartphones (iPhone 3GS, iPhone 4S, and HTC Desire). Participants were asked to complete an electronic version of the SUS (available in Dutch and English) on an iPad and to select six cards from the ReactionDeck toolkit, available on computer, to describe the hedonic quality of the app.

## Results

### Results of the Expert Evaluation

The first search process resulted in a full list of 61 apps. In the eligibility testing phase, 15 apps were excluded because they were specifically focused on pediatric/newborn life support, on CPR for animals, or were listed but not downloadable. The remaining 46 apps were evaluated by experts.

While all 46 evaluated apps offered training features, only 75% (35/46) included emergency (real incident) support and 35% (16/46) followed current ERC/AHA guidelines [5,6]. In total, 28% (13/46) of available CPR apps fulfilled the three minimum criteria: training feature, conformity with ERC/AHA 2010 BLS Guidelines, and emergency (real incident) support (Table 2).

Of the 46 apps from the experts’ evaluation, 15% (n=7) offered direct access to an emergency call as well. The only app that offered an accelerometer for real-time feedback during compressions is Pocket CPR; FDNY Lifesaver Beta V1.0 offered GPS location.

**Table 2:** CPR apps and features for layperson evaluation.

App name	Mandatory features	Important feature	Special features
CPR & Choking, by University of Washington and King County EMS	yes	yes	compression-only CPR
CPR Steps (now available as Free CPR), Evolving Monkeys, LLC	yes	yes	no
Emergency First Aid &Treatment Guide, by Phoneflips	yes	no	compression-only CPR
FDNY Lifesaver Beta V1.0, by the New York City Fire Department/Bavelle Technologies	yes	no	compression-only CPR
First Aid White Cross, by Bruno Mandolesi	yes	yes	no
Hands-Only CPR, by the American Heart Association/Jive Media Inc.	yes	yes	compression-only CPR
Leben retten, by the German Heart Foundation/Fuse GmbH	yes	yes	compression-only CPR
Pocket CPR, by Bio-Detek, Inc.	yes	yes	focus on compression-only CPR
			use of accelerometer
Pocket First Aid & CPR, by the American Heart Association/Jive Media Inc.	yes	no	compression-only CPR
Reanimatie, by the Dutch Heart Foundation	yes	yes	no
SCDF Choking CPR AED, by The Singapore Civil Defence Force (SCDF)	yes	no	no
SOS American Red Cross	yes	yes	compression-only CPR
St John Ambulance First Aid, by St. John Ambulance	yes	no	compression-only CPR

### Results of Layperson Evaluation of Usability and Hedonic Quality

#### Demographics

The 13 apps providing the minimum criteria were included in this second phase.

A total of 14 volunteers were recruited (5 female); 7 participants had little experience with mobile apps, while the others had moderate or much experience. Of the 14 participants, 9 had no experience with CPR, 2 had taken a first aid course once, and 3 had dedicated CPR training. Two participants were in the age range 20-29 years, 3 in the age range 30-39 years, 2 between 40-49 years, and 7 participants were above 50 years of age. All participants had good English language skills, all but one were Dutch native speakers, and most participants could understand German well.

#### Usability Evaluation

To test agreement for the usability evaluation, the ICC was calculated for the two directions of the SUS scale. This analysis has resulted in strong to perfect agreement: ICC1=.83, *P*<.001, 95% CI 0.75-0.882 and ICC2=.79, *P*<.001, 95% CI 0.695-0.869. Table 3 shows the results of the usability evaluation. For the five ratings per app, mean values were calculated. Items 4 and 10 were taken as a subscale for learnability, while the rest of the items contribute to the construct usability. The study shows that only five apps have a usability score above average (SUS>68) and fall into the percentile rank of above 50%.

**Figure 3:**
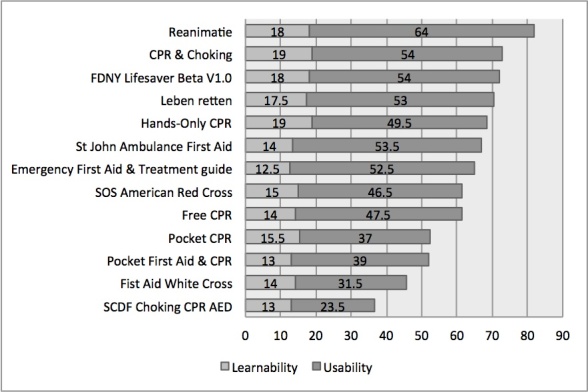
Usability evaluation results with subscales learnability and usability.

We furthermore analyzed results with regard to subscales “learnability” and “usability”. Results of this analysis are presented in Figure 3.

This additional perspective on the data shows that most apps with high usability also have high learnability. Only for the Hands-Only CPR app was the learnability evaluated with one of the highest values while the usability subscale delivers resulted below average.

**Table 3:** Mean System Usability Scale (SUS) score and standard deviation.

App name (language)	Mean SUS score	SD	Percentile rank, %
Reanimatie (Dutch)	82.00	14.40	92.6
CPR & Choking (English)	73.00	11.91	66.5
FDNY Lifesaver Beta V1.0 (English)	72.00	14.51	63.1
Leben retten (German)	70.50	19.48	58.1
Hands-Only CPR (English)	68.50	15.17	51.6
St. John Ambulance First Aid (English)	67.00	23.48	46.9
Emergency First Aid & Treatment Guide (English)	61.90	8.50	33.1
Free CPR (aka CPR Steps) (English)	61.50	14.43	32.1
SOS American Red Cross (English)	61.50	16.55	32.1
PocketCPR (English)	53.80	21.70	17.8
Pocket First Aid & CPR (English)	52.00	25.46	15.4
First Aid White Cross (English)	45.50	21.97	9.1
SCDF Choking CPR AED (English)	36.50	11.67	4.3

### Results of Hedonic Quality Evaluation

For hedonic quality evaluation, Table 4 presents adjectives selected more than once.

**Figure 4:**
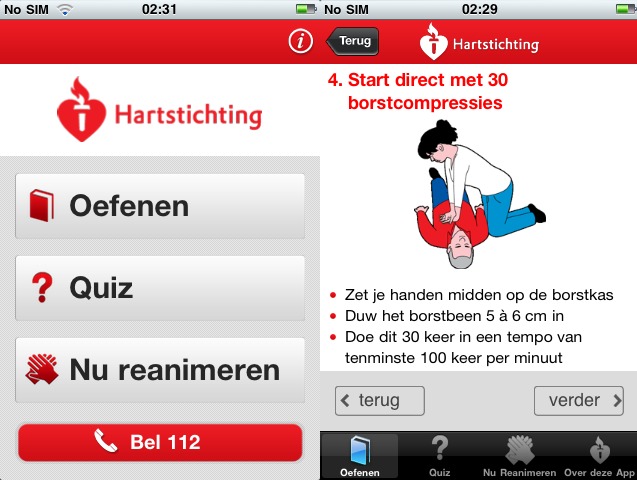
Screenshot Reanimatie app by the Dutch Heart Foundation.

**Figure 5:**
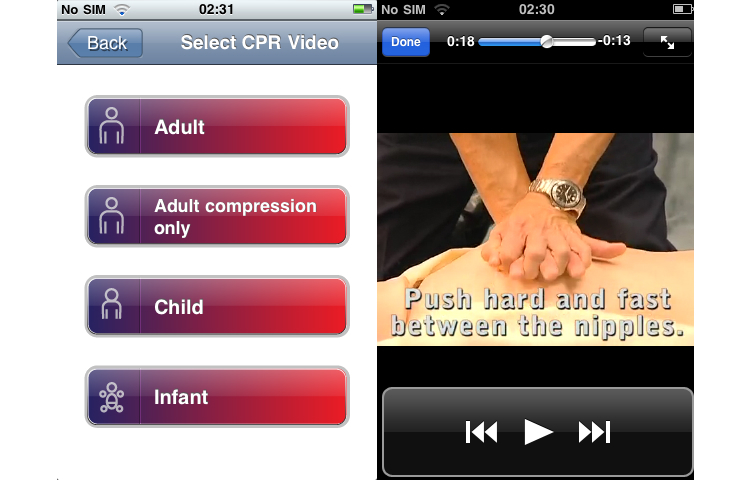
Screenshot CPR and choking by University of Washington and King County EMS.

**Figure 6:**
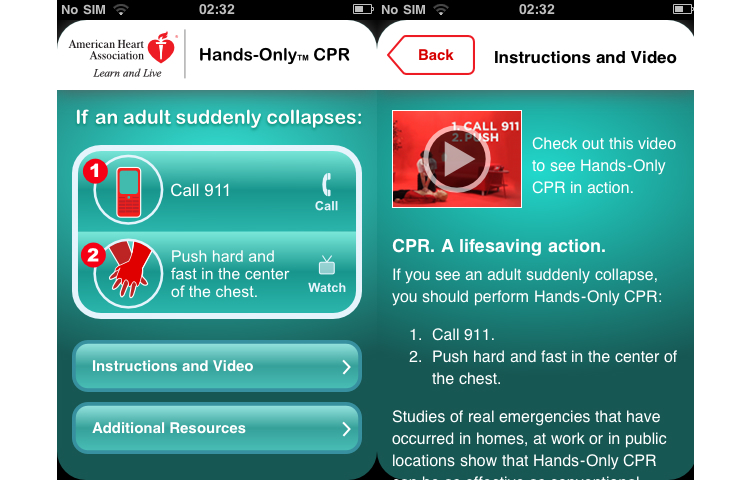
Screenshot Hands-only CPR by the American Heart Association/Jive Media Inc..

**Figure 7:**
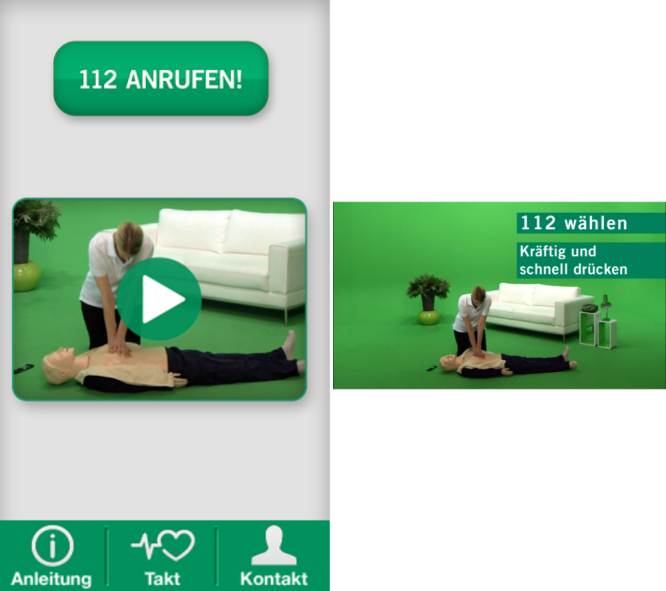
Screenshot Leben retten app by the German Heart Foundation/Fuse GmbH.

The hedonic quality evaluation delivers mixed results: one app (Reanimatie, Figure 4) ranked high in the usability evaluation and received very positive results for hedonic quality as well. Other top-ranked apps received positive adjectives in most instances but also some relevant negative adjectives (see Table 3) (eg, CPR & Choking shown in Figure 5 and FDNY Lifesaver Beta V1.0). The two other top-ranked apps from the usability evaluation (Hands-Only CPR shown in Figure 6 and Leben retten in Figure 7) received mainly negative hedonic adjectives. Other apps received mixed results in this part of the evaluation.

**Table 4:** Hedonic quality results with adjective occurrences >1.

App name	Hedonic quality adjectives
Reanimatie	Professional, effective, easy to use, inviting, stable
CPR & Choking	Simplistic, ordinary, understandable, ineffective, easy to use, dull, usable
FDNY Lifesaver Beta V1.0	Helpful, usable, relevant, useful, dull, poor quality
Leben retten	Understandable, dull, simplistic, ineffective
Hands-Only CPR	Unattractive, easy to use, simplistic, poor quality
St. John Ambulance First Aid	Slow, useful
Emergency First Aid & Treatment Guide	Understandable, reliable
Free CPR (aka CPR Steps)	Relevant, dull, simplistic, ineffective, easy to use
SOS American Red Cross	Understandable, usable
PocketCPR	Frustrating, difficult, confusing, hard to use
Pocket First Aid & CPR	Ineffective, intuitive, helpful, professional, usable
First Aid White Cross	Annoying, ineffective, frustrating, dull, hard to use, slow, poor quality
SCDF Choking CPR AED	Hard to use, undesirable, slow, complex, annoying, helpful, ineffective, unattractive

## Discussion

### Principal Results

This study emphasizes that several apps are available for resuscitation training and real incident support on the two largest app markets. However, very few are designed according to current CPR guidelines and offer acceptable usability as well as hedonic quality for laypeople. At the time of this study, only about five apps could be recommended in terms of guidelines and usability. In the 2010 AHA/ERC guidelines, depth and rate of chest compressions were raised. The chance of survival after a cardiac arrest is inevitably linked to high-quality chest compressions. Therefore, it is extremely important to comply with current guidelines [5,6]. Many available apps do not meet these basic requirements and, in the worst case, an app might inhibit optimal chest compressions.

In a recent systematic review, Mosa et al analyzed effects of health care applications for smartphones [7]. While this review delivered interesting findings and a good overview of existing studies, only 83 mobile applications were included in their review out of the approximately 20,000 mobile applications in the medicine category and 44,000 apps in the “Medicine” and “Health and Fitness” categories on the Apple App Store. This large number of available mobile apps has motivated van Velsen et al to address the problem of “app overload” [8]. While the authors propose to centralize the development of mobile apps for health and medicine, we think that quality assurance mechanisms are the more appropriate solution to address the large number of mobile apps. This approach is in line with earlier proposals to deal with quality management of medical information on the Internet [23] and also recent guidance by the US Food and Drug Administration about the regulation of mobile medical applications [24].

The mixed-methods evaluation conducted in this study and its results are a first step to optimize the development and evaluation of mobile apps for resuscitation training and real incident support. Particular focus is on content as well as usability and hedonic quality. Furthermore, it supports the informed selection of mobile apps for training situations and/or real incident support.

During the initial screening phase, several CPR-related apps that did not fulfill the inclusion criteria of this study attracted our attention because of very promising approaches. PulsePoint, for example, uses the Global Positioning System (GPS) to locate potential responders in the vicinity of an emergency and directs them to the place of action. Fatal no-flow time in a cardiac arrest might be reduced [25].

Low et al as well as Semeraro et al showed increased CPR performance by health care professionals and layperson end-users, respectively, in simulated medical emergency cases. Feasibility in and relevance for real emergency cases is not proven yet and should be of interest for further investigations [25,26].

### Limitations

Our study has several limitations. CPR apps were first screened for inclusion criteria by only two board-certified emergency physicians. Thus, one could argue that some apps may have been misjudged, but two out of three mandatory criteria directly reflect the enquiry (potential support for training and real incidents). The third criterion, current ERC/AHA BLS guidelines, represents generally accepted European and American standards and this appraisal is quite easy for an expert physician. As the “important” and “desirable special features” constitute a more subjective expert view, these did not lead to an exclusion from the evaluation. Furthermore, we calculated the interrater reliability (substantial for the experts) to ensure that decision making by two experts delivers adequate agreement. We therefore consider this approach as a valid way to include and categorize apps for this study. In future studies or when setting up more detailed quality standards, it is probably reasonable to include more experts.

A limitation resulting from the apps is that three out of five recommended apps are available only in English (CPR & Choking, FDNY Lifesaver Beta V1.0, and Hands-Only CPR), one only in German (Leben retten), and one (Reanimatie) only in Dutch. Since we evaluated the apps at a Dutch University, there might have been a language bias that led to the very high usability value of the “Reanimatie” app.

Adherence to current CPR guidelines and usability for the public was the main focus in this study. Hence, conclusions for developing and selecting apps can be drawn. However, we cannot make conclusions about the efficacy of each single app in a real emergency situation. The use of the ReactionDeck toolkit served the purpose of collecting input from study participants about the hedonic quality of the mobile apps. However, this method has not been further evaluated regarding its reliability and validity for analyzing hedonic aspects of software.

In the digital age, especially when working on mobile applications, timeliness of data is a general problem. The number and quality of apps are constantly changing. In addition, lists of apps are likely to be incomplete due to varying availability of apps in different stores (operator-related as well as country-related), varying search methods, or apps published after the market search for this study. Therefore, it remains unclear which apps are “the best ones” for CPR support and training at the time of publication. We must highlight the enormous number of apps that are not useful or have substantial deficits as a key finding. The smartphones themselves also have an impact on the appearance and usability of an app and therefore may have affected the participants’ experiences.

### Further Research

Smartphones and easy-to-learn approaches like “compression-only CPR” seem to match well: situational support and easily accessible information (ie, apps) can be provided in all situations. Often-mentioned reasons for low rates of bystander CPR (eg, fear and lack of knowledge) might be alleviated by these supporting devices resulting in more laypeople being motivated to perform CPR. In contrast, teaching obsolete guidelines or giving too detailed information could deter the public from performing CPR or lead to worse CPR quality.

Recently, You et al proposed the use of quick response (QR) codes displayed in public places and on personal belongings like key rings, wallets, and necklaces of patients with cardiovascular risk to provide access to critical video instructions required during resuscitation [27].

The top-ranked apps of this study, as well as apps released after the evaluation period (eg, Lifesaver Mobile, Viva! CPR, Staying Alive 3D), characterize the evolution from simple teaching materials to multifunctional programs with feedback devices (eg, metronome) and game-based learning modules for virtual scenarios and/or real incidents. Future research will need to focus on analyzing more closely which features motivate end-users to use these apps for training, refreshment of knowledge, and real incident support and which features are most effective. While recently published apps invest more and more in professional media production or 3D environments, it is questionable whether these huge investments also have an impact on increasing knowledge, skills, or the willingness to help in a real cardiac arrest situation.

### Conclusions

To our knowledge, our study is the first to give a general overview of existing apps for resuscitation training and real incident support. All apps were examined under consideration of current CPR guidelines as well as usability and hedonic quality for layperson end-users. This study has shown availability of a multitude of mobile apps for CPR training and real incident support in the largest mobile app markets. Unfortunately, only a few follow recent guidelines, are designed with acceptable usability, and are easy to learn for non-expert users. While mobile phones are increasingly integrated into the chain-of-survival, the wide usage of mobile apps for resuscitation training and real incident support cannot be recommended without caution at this point of time. More interdisciplinary studies and joint development of mobile apps for resuscitation training and support are needed. Besides correct guidelines and good usability, testing should include efficacy in real incident scenarios wherever possible. The method used in this study has the potential to be applied to other evaluation studies where a focus on both regulation and end-users is required for quality assurance of mobile apps in the health context.

## Abbreviations

AHA: American Heart Association
BLS: Basic Life Support
CPR: cardiopulmonary resuscitation
ECC: external chest compression
ERC: European Resuscitation Council
ICC: intraclass correlation coefficient
ISO: International Organization for Standardization
SUS: System Usability Scale
